# Barriers to employment of Australian cancer survivors living with geographic or socio‐economic disadvantage: A qualitative study

**DOI:** 10.1111/hex.13238

**Published:** 2021-04-07

**Authors:** Emma Kemp, Vikki Knott, Paul Ward, Suzana Freegard, Ian Olver, Julia Fallon‐Ferguson, Jon Emery, Chris Christensen, Monique Bareham, Bogda Koczwara

**Affiliations:** ^1^ College of Medicine and Public Health Flinders University Adelaide Australia; ^2^ Flinders Centre for Innovation in Cancer Bedford Park Australia; ^3^ Australian College of Applied Psychology Brisbane Australia; ^4^ Menzies School of Health Research Brisbane Australia; ^5^ University of Adelaide Adelaide Australia; ^6^ School of Primary, Aboriginal, and Rural Health Care University of Western Australia Perth Australia; ^7^ Department of General Practice and Centre for Cancer Research Faculty of Medicine, Dentistry and Health Sciences University of Melbourne Melbourne Australia; ^8^ Cancer Voices South Australia South Australia Australia; ^9^ School of Health Sciences University of South Australia South Australia Australia; ^10^ NHMRC Centre of Research Excellence in Cardiovascular Outcomes Improvement Curtin University Perth Australia

**Keywords:** cancer survivors, employment, return to work, social determinants of health, vulnerable populations

## Abstract

**Background:**

Opportunities for cancer survivors’ employment can both reflect and perpetuate health inequities, as employment is an important social determinant of health. Socio‐economic and geographic disadvantage is associated with greater difficulty finding work, but little is known about work needs of Australian cancer survivors living with disadvantage.

**Objective:**

This study examined survivor and health‐care professional (HCP) perspectives on barriers experienced by Australian cancer survivors experiencing disadvantage when attempting to remain at or return to work.

**Method:**

Focus groups and individual interviews were held with cancer survivors (N = 15) and oncology and primary HCPs (N = 41), focusing on communities at risk of disadvantage. Participants were asked about employment barriers and facilitators in general and in the context of disadvantage. Themes were identified using framework analysis.

**Results:**

Geographic and socio‐economic disadvantage resulted in specific individual‐ and system‐level barriers. These related to distance from treatment and support services and limited availability and suitability of work for survivors living with geographic disadvantage, and limited availability, security, and flexibility of work and previous unemployment for survivors living with socio‐economic disadvantage. Identified needs included system‐level changes such as public and workplace‐level education, legislative and policy changes, and better access to resources.

**Conclusions:**

Cancer survivors living with disadvantage experience limited access to flexible employment opportunities and resources, further perpetuating their disadvantage. Promotion of health equity for cancer survivors living with disadvantage requires systemic changes to support attempts to remain at/return to work.

**Patient or public contribution:**

This study included cancer survivors and HCPs as investigators, authors and participants.

## 1 INTRODUCTION

Support regarding employment and remaining at or returning to work represents an important unmet need for cancer survivors, their health‐care professionals^1^ and employers.^2^ Cancer survivors are approximately 1.4 times more likely to be unemployed than healthy controls^3^ and are delayed in return to work or retire early.^4^ Employment has substantial individual benefits for physical, psychological and social health, including sense of personal worth and identity;^5^ self‐esteem;^6^ distraction;^2^ better physical and psychosocial functioning;^7^ and sense of normalcy and social connectedness.^8^ In contrast, loss of employment can result in poorer quality of life and reduced psychosocial functioning,^9^ and has significant negative financial implications.^10^, ^11^ As employment is recognized as an important social determinant of health^12^, ^13^ impacting on health outcomes for cancer survivors,^14^ the ability to remain at or return to work is important for health promotion and equity. Difficulty remaining at or returning to work during or after cancer treatment is attributable to impaired health; functional limitation; work demands; policy and procedures; and economic factors.^15^ Work outcomes are influenced by discrepancies between work demands and individual capabilities,^15^ with return to work negatively impacted by physical/manual work demands, lower education and lower income.^16^, ^17^ Reviews suggest a coordinated approach to return to work involving health and vocational professionals, with more research on environmental and occupational factors influencing the return to work.^18^

Survivors living in rural and socio‐economically disadvantaged communities may face additional challenges related to employment, compounded by the work demands of available occupations and by socio‐economic factors. A study in the United States found rural cancer survivors were less likely to return to work than metropolitan‐ or urban‐dwelling survivors, with rural survivors 66% more likely to retire early compared to urban survivors of similar age, education and cancer stage.^19^ A European study found cancer survivors from lower socio‐economic groups were less likely to return to work and took longer to return or to regain employment than survivors from groups experiencing less socio‐economic disadvantage, despite routine offering of occupational rehabilitation to cancer survivors.^20^ Authors of these studies argue the employment needs of rural cancer survivors and those living with socio‐economic disadvantage are distinct from the needs of other cancer survivors, due to the nature of occupations prevalent in these groups.^19^, ^20^ This is consistent with findings indicating that the employment gap for cancer survivors is larger for those employed in agriculture, forestry, fishery, transport, manufacturing and services, compared with those in sciences, humanities, administrative, managerial or clerical work.^21^ Therefore, approaches aiming to assist cancer survivors living with disadvantage to remain at or return to work must consider the potential for these survivors’ distinct needs to impact upon this process.

Little is known about challenges work participation for cancer survivors living with disadvantage in Australia. The Australian Bureau of Statistics defines relative socio‐economic advantage and disadvantage in terms of access to material and social resources, and their ability to participate in society. Socio‐Economic Indexes for Areas (SEIFA) indicates a trend for greater disadvantage in outer metropolitan, regional and remote areas.^22^ Socio‐economic disadvantage relates to higher cancer rates, worse cancer outcomes and additional employment challenges,^23^ due to low population density and limited opportunities for employment or vocational rehabilitation.

A previous study exploring Australian cancer survivors’ return to work recommended including the perspectives of primary care health professionals, due to the integral role they may play in facilitating return to work.^1^ Primary care perspectives are particularly important given recent recommendations for expanding primary care involvement in cancer survivorship care, including management of late effects of cancer treatment, and providing informational and psychological support,^24^, ^25^, ^26^ all of which are relevant to employment. In geographically and socio‐economically disadvantaged communities, where access to specialist and allied health services tends to be more limited,^27^, ^28^, ^29^ the role of primary care professionals in facilitating remaining at or returning to work may be even greater. A previous review has also indicated employment barriers and enablers operate at the systemic level (eg organisation, policy); thus, combining individual and systems perspectives allows for a broader scope to address employment barriers.^15^ A more comprehensive exploration of return to work experiences of Australian cancer survivors living with either geographic or socio‐economic disadvantage can be achieved by exploring the perspectives of survivors along with perspectives of primary health care and oncology care professionals, and by examining both individual and systemic issues from survivor and HCP perspectives.

This study therefore aims to examine perspectives of cancer survivors and primary and tertiary health‐care professionals (HCPs) on unique individual and system‐level barriers and needs experienced by Australian cancer survivors living in disadvantaged circumstances when attempting to remain at or return to work.

## 2 METHOD

### 2.1 Participants and procedure

Ethics approval for this study was obtained from Southern Adelaide Clinical Human Research Ethics Committee. Two cancer consumers (defined as ‘a person affected by cancer as a patient, survivor, carer or family member’,^30^ in this case both cancer survivors) were involved in study design, analysis and writing and co‐facilitated focus groups but did not contribute their perspectives to the data.

Qualitative, inductive methodology (using semi‐structured interviews and focus groups) was used to explore the experiences and perceptions of cancer survivors and HCPs, with particular focus on barriers and enablers to remaining at/returning to work. This approach (using both semi‐structured interviews and focus groups) allowed for explorations and discussions of relevant experiences and perceptions of participants, in addition to creating an atmosphere conducive to an open and uninhibited flow of conversation.^31^ In this way, the interview process and data analysis recognized that ‘return to work’ may (or may not) have been an issue for the cancer survivors and HCPs in the study (as opposed to predetermining its relevance). This enabled focus group and interview facilitators to draw out the world‐views of the participants and limited the influence of researchers’ preconceptions.

All participants completed informed consent prior to participating. Participants were sampled using a non‐probabilistic, purposeful sampling method to achieve sampling via relevance.^32^ The sample was structured primarily by socio‐economic status and rural location, since these groups were assumed to incur the double‐edged sword of having higher rates of cancer and lowest population‐level employment rates.^23^ Participants were therefore recruited from rural and outer metropolitan areas with a high prevalence of socio‐economic disadvantage and evidence of poorer cancer outcomes, identified using the Atlas of Cancer in South Australia.^33^ Cancer consumers and HCPs from medical, nursing and allied health disciplines were invited to participate in focus groups or telephone interviews; the option of either focus groups or interviews was offered to accommodate for participant circumstances (location, commitments, practice schedules), convenience and preferences. Invitation was facilitated by emails sent by the research assistant and investigators to consumer organizations, clinical groups and general practices in rural and outer metropolitan areas characterized by higher levels of disadvantage. Interviews and focus groups followed a topic guide (see Table 1 for example questions and Appendix 1 for full topic guide). All focus groups and interviews were facilitated by researchers experienced in conducting qualitative research and with clinical experience in managing distress in cancer survivors. Interviews were facilitated by these same researchers or by a social science researcher with training in qualitative research and in managing participant distress. All participants were advised that if they experienced distress due to participation, they could skip questions or immediately withdraw and would be offered support free of charge.

**TABLE 1:** Example of focus group/interview questions on remaining at/returning to work after cancer in disadvantaged communities

1. What in your opinion are patients’ expectations regarding work after cancer before they commence cancer treatment?
2. How do you think they change, if at all, after they experience cancer treatment?
3. What is your view of the impact of cancer and its treatment on current and future work?
4. Are there any aspects to these experiences that are unique to patients living in rural/remote Australia?
5. Are there any aspects to these experiences that are unique to patients living in communities where employment is limited?
6. What do you see as the barriers to returning to work?
7. Who do you feel would be best to address these barriers? How could they help?
8. What supports and resources relating to employment would you like to have access to?

### 2.2 Analysis

Interviews and focus groups were transcribed and then analysed using the software program NVivo 12 according to the ‘framework method’ of thematic analysis.^34^ This method facilitates qualitative analysis that is directed a priori by a specific research objective but remains responsive to emergent themes within the data. Thus, a coding framework was developed based on key concepts from the research questions, but codes also were generated inductively to capture unexpected concepts. A preliminary coding framework was developed from a subset of transcripts and then developed iteratively through discussion with investigators and analysis of the complete set of transcripts to produce the final coding framework. This framework was applied across all survivors and HCPs, to enable comparisons and to facilitate a comprehensive picture of issues raised. Separate coding frameworks were not generated for survivors and HCPs, given the potential for similar issues to be recognized by these groups, albeit from differing perspectives.

## 3 RESULTS

### 3.1 Participants

A total of 56 adults (15 consumers, 41 HCPs) participated in individual or paired interviews, or focus groups (Table 2), resulting in 16 transcripts. Detailed individual demographic data were not collected, to minimize burden on participants living/working with disadvantage and avoid concerns regarding identification in areas with low population density. Information on socio‐economic disadvantage was characterized at focus group level using the Socio‐Economic Indexes for Areas (SEIFA) Index of Relative Socio‐economic Advantage and Disadvantage (IRSAD), which characterizes areas in quintiles, from quintile 1 = most disadvantaged to quintile 5 = most advantaged^22^ (Table 2).

**TABLE 2:** Interview and focus group participants—gender and IRSAD quintile

Cancer survivors	n (n female)	IRSAD quintile
Individual interviews	8 (6)	
Paired interview (Remote)	2 (2)	Quintile 1
Consumer focus group (Regional)	5 (5)	Quintile 1

Health‐care professionals	N (n female)	IRSD quintile
Primary care focus group 1 (Regional)	7 (4)	Quintile 3
Primary care focus group 2 (Remote)	6 (3)	Quintile 1
Primary care focus group 3 (Regional)	6 (5)	Quintile 2
Primary care focus group 4 (Regional)	5 (3)	Quintile 1
Primary care focus group 5 (Outer metropolitan)	8 (6)	Quintile 1
Treatment centre focus group 1 (Metropolitan)	9 (6)	Quintile 1
Total participants	56 (40)	

### 3.2 Barriers/enablers of remaining at/returning to work after cancer treatment

**FIGURE 1:**
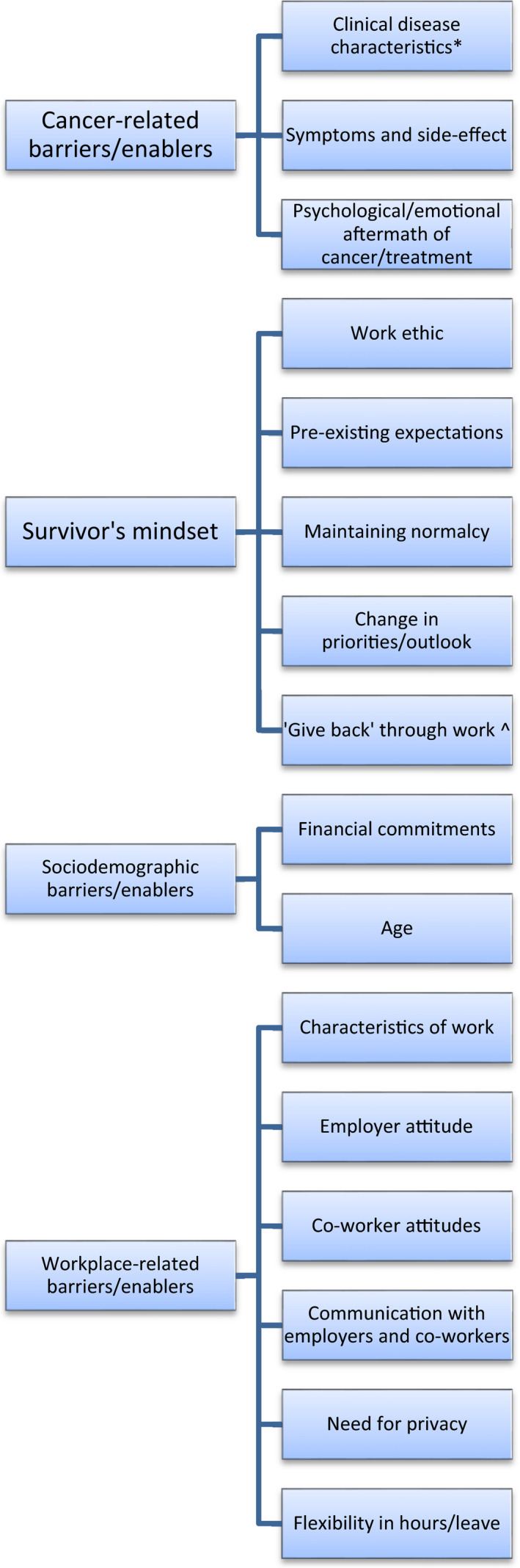
Individual‐level barriers/enablers in remaining at/returning to work (general). *Identified only by health‐care professionals; ^identified only by consumers. (All other themes were identified by both groups.)

**FIGURE 2:**
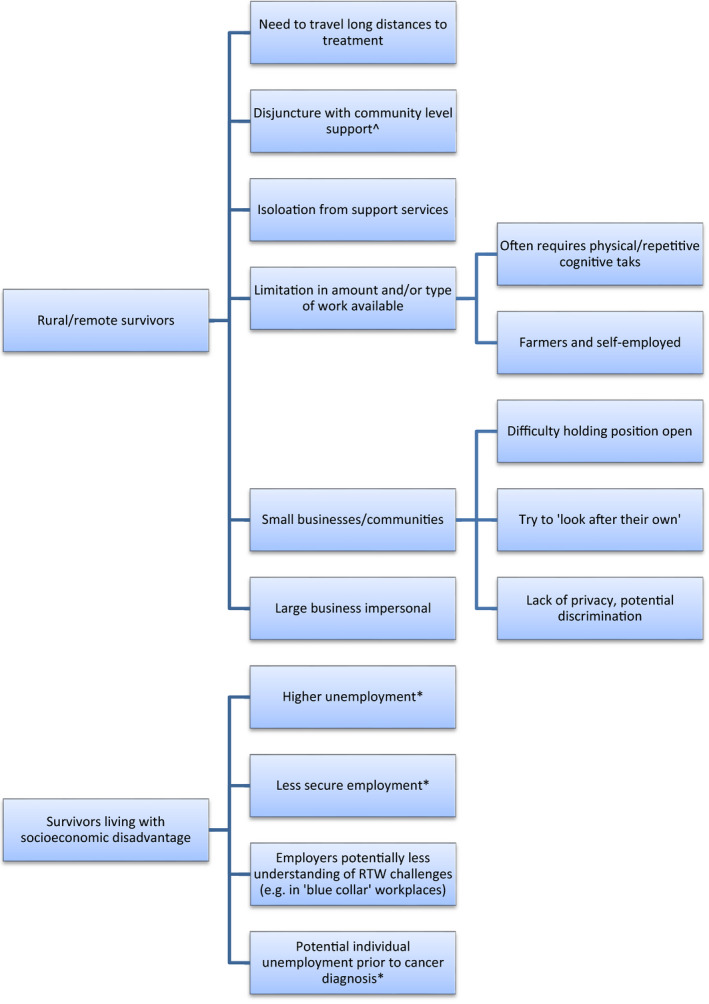
Additional issues experienced by disadvantaged survivor groups. *Identified only by health‐care professionals; ^identified only by consumers. All other themes were identified by both groups

**FIGURE 3:**
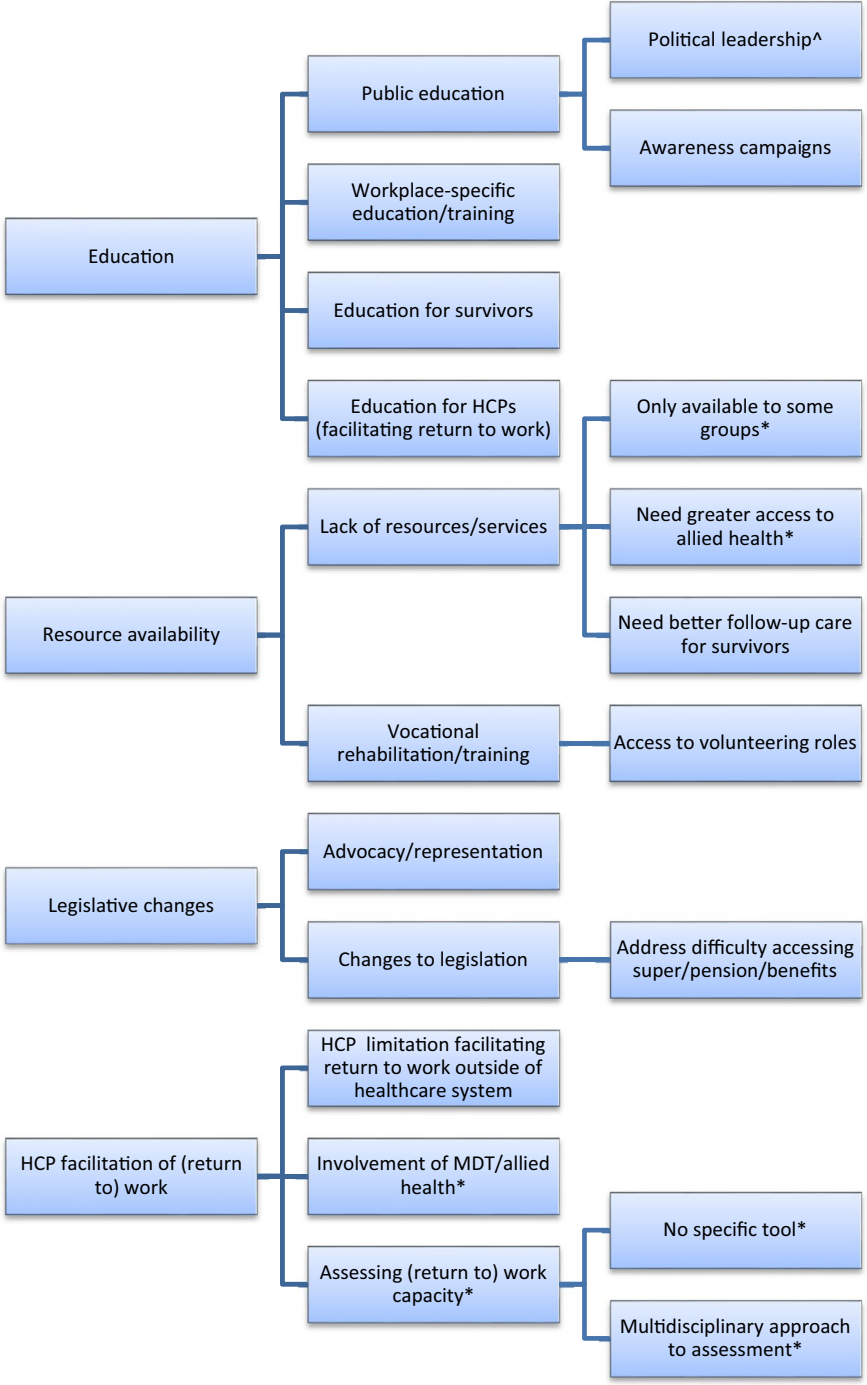
Systemic issues and solutions to remaining at/returning to work. *Identified only by HCPs. ^Identified only by survivors. All other themes were identified by both groups

Themes in barriers and enablers of remaining at/returning to work during/after cancer treatment were identified by survivors and HCPs on three levels: (1) individual‐level barriers/enablers experienced by cancer survivors in general (Figure 1), (2) specific subsets of individual‐level barriers/enablers experienced by cancer survivors who are (a) rurally residing or (b) living in socio‐economically disadvantaged circumstances (Figure 2) and (3) system‐level barriers/enablers affecting cancer survivors (Figure 3) (for examples of all themes and subthemes, please see Appendix 2).

### 3.3 Individual‐level barriers/enablers experienced by cancer survivors in general

Individual‐level barriers/enablers identified by survivors and HCPs included cancer‐related, ‘mindset’, sociodemographic and workplace‐related factors.

***Cancer‐related barriers/enablers*** experienced on an individual level included *clinical disease characteristics* such as type and severity of cancer and required treatment, and the presence (as a barrier) or absence (enabler) of *symptoms and side‐effects* of cancer or treatment.

It was difficult. ….. I just felt exhausted and sick and what have you. But once the pain eased up which was about halfway through the treatment, I was able to continue and my leave taking wasn't too bad. (Survivor interview 2).

Cancer‐related barriers also included *psychological and emotional aftermath of cancer/treatment*. This included anxiety, fear, or panic, changes in work‐related confidence/self‐efficacy, changes to self‐image, and difficulty getting into a work ‘headspace’.

Just those little things, waking up early, catching the bus again, going – sitting for a long period of time, interacting with people, having responsibilities, all of those, which is normal in the job. When you’ve been off for so long and you’ve had such a very traumatic experience it’s difficult. (Survivor interview 8).

***Survivor's mindset*** or attitude towards work and life priorities was an additional theme encompassing a number of psychological barriers/enablers to remaining at/returning to work. These included *work ethic*, *pre‐existing expectations* of an easier return to work or of not being able to return; wanting to work as a distraction or to *maintain normalcy*; not wanting to work due to a *change in priorities/outlook*; and being inspired through the cancer experience to ‘*give back’ through work*.

Some people just want to put it to one side and get on with it, so I guess their work ethic or how they deal with difficult situations comes into it as well. (Primary care focus group 1).

***Sociodemographic barriers/enablers*** to remaining at/returning to work included *financial commitments*, which were seen as increasing motivation to remain at/return to work.

If you’re in the age group that you’re still paying off your mortgage and you’ve got children as dependents, then you’re more likely to need to work, I guess, regardless of ability necessarily. (Treatment centre focus group).

For some, financial necessity of remaining at/returning to work was offset by availability of income protection insurance, a pension or benefit, or their partner's income.

*Age* was identified as barrier/enabler that affected likelihood of retirement (those closer to retirement age were considered less likely to remain at/return to work), fatigue levels, and employment and retraining opportunities.

People 50 to 65 I think are particularly at risk because cancer is prevalent, starts to become prevalent in this age group. They’re already at risk in terms of maintenance of work, and they’re in a black spot in terms of access to forms of financial support, so they feel up against the wall about going back to work, they just don’t have the energy. They can’t retrain. (Survivor interview 7).

***Workplace‐related barriers/enablers*** included *characteristics of work*. Barriers included the requirement of manual/physical labour, high levels of concentration, or dealing with others’ crisis situations.

I worked with dementia residents and pushing around wheelchairs taking the weight of residents, then being able to assist with manual handling and then thinking I may not be up to it When I had a PICC [peripherally inserted central catheter] line in my arm I was informed they had a weight limit which also had to be factored in. (Survivor interview 8).

Further workplace‐related barriers/enablers included *employer attitude*, with a more understanding or flexible employer being an enabler and less understanding being a barrier. More supportive *co‐worker attitudes* were identified as an enabler and co‐worker discrimination/lack of understanding was a barrier.

When [I was] diagnosed they asked, “How long would you like off?” Not knowing, I said at least 6 months. They took on someone on a contract. When the 6 months ended, they asked again, and I said another 6 months. They were great with that. I had a phenomenal boss, who said I had great potential. Well, if that doesn't help you get better, I don't know what does. (Survivor interview 8).

*Communication with employers and co‐workers* facilitated employer and co‐worker understanding. However, this was offset by the *need for privacy and risk of discrimination* from either employers or co‐workers if cancer was disclosed.

For my first employer it was okay, because they knew my situation. But I, for my next roles ‐ because I understood my limitations, I wouldn’t disclose all of that. I did once, and that worked against me, so to disclose my limitations was ‐ well I wouldn’t do it in the end. (Paired survivor interview).

Further workplace‐related barriers/enablers included *flexibility in hours and available leave* (to attend appointments or get through period of severe symptoms/side‐effects).

I was never offered to come back part time or to come back as a test run and subsequently I guess I kept getting ill or very low in energy and suffering complications from the surgery that kept having me readmitted to hospital. (Survivor interview 6).

### 3.4 Additional issues experienced by disadvantaged survivor groups

Both survivors and HCPs identified additional issues experienced by disadvantaged survivor groups attempting to remain at/return to work. ***Issues experienced by rural/remote survivors*** in remaining at/returning to work included the *need to travel long distances to treatment centres*. This was identified as impacting on availability and ability to work.

What would be unique to the farmers in rural and remote areas is distance to travel to appointments. That’s very hard to do so you try to condense them all in one day…But some people are so stuck that they’ve got to go back to work the next day. They get up early, 5 o’clock in the morning to get to an appointment and get home at 9 or 10 o’clock at night and go to work the next day. (Survivor interview 4).

Travel to distant treatment centres was also noted to lead to a *disjuncture with community‐level support* that could otherwise facilitate return to work, such as local cancer support groups, along with *isolation from services* that might assist with recovery/return to work, including transport (for travel to treatment, support services or workplaces).

A lot of our residents, they are sent through to Adelaide. The GP organises it. Off they go. They come back here, and they know nothing…So when they come back here, they are really so isolated. (Consumer focus group).

Rural/remote survivors were also affected by *limitations in the amount and/or type of work available*.

… there's no work. So, if you do actually lose your job it's very hard to find another one, particularly in your field of expertise. (Primary care focus group 2).

Types of work available in rural locations were identified as *often requiring manual/physical or repetitive cognitive tasks* that may present issues for those suffering from treatment side‐effects.

And being physical a lot of it's ‐ it's physical work in the mills now, but a lot of it's computerised. So, you've got to really be hands‐on with your head and that doesn't ‐ chemo and all that doesn't do your head any good after 10 hours a day working. (Consumer focus group).

*Farmers and those self‐employed* in small businesses were recognized as being in particularly difficult situations.

They’re farmers or they’ve got stock and animals to worry about. So, they can’t just up and retire or finish, there’s a lot more to consider. (Treatment centre focus group).

Several participants also noted the prevalence of small businesses as employers in rural areas and the difficulty of these businesses in keeping positions open for survivors looking to return to work. On the other hand, larger organizations were considered potentially unsupportive due to high unemployment and a casual workforce.

Because there’s such high unemployment, they’ve got a huge pool of people. But if you’re sick and you can’t do the job, because you can’t be in, say, a chicken farm where there’s feathers and dust and stuff…if you had lung cancer, you wouldn’t be able to work there, so they would say, “No, there’s no job here because we can’t have a claim against us for compensation if you get sick, when you’ve come with a pre‐existing condition.” (Survivor interview 4).

In contrast, small businesses and the rural community were seen as supportive and more likely to try to ‘look after’ employees.

The good is that because we're a small community, people tend to care for each other, which is nice. So, you know, employers will be inclined to try and give them work back. (Primary care focus group 2).

However, small communities also meant a potential lack of privacy and consequent increased opportunities for discrimination.

I applied for quite a few jobs, but I couldn’t secure an interview. I put that down to perhaps they were concerned because I had the cancer tag… I don’t know, no one’s going to say that to your face or tell you. (Paired survivor interview).

***Issues experienced by survivors living with socio‐economic disadvantage*** in remaining at/returning to work included *higher unemployment* and *less secure employment*, meaning less flexibility in hours/ability to take leave and consequently, less ability to remain at or return to work during or after treatment.

These patients are generally more inclined to want to get back to work quicker because they’re feeling like their job’s threatened. If they take any more time off, they’re going to lose their job, there’s a lot of pressure, from what I’ve seen anyway. (Treatment centre focus group).

Some participants also considered that *employers* of cancer survivors living with socio‐economic disadvantage might have *less understanding of return to work challenges* than employers of less disadvantaged survivors. Others related how lack of understanding could contribute to lack of flexibility.

If say it’s an area where it’s difficult to find a job and a lot of jobs are casual or I guess blue collar jobs, it might be more difficult to even I guess find understanding in that context than it would be for example if you worked in an area where you were with a bunch of health professionals. (Treatment centre focus group).

Some participants felt RTW difficulty could be exacerbated for survivors who were experiencing, and arguably accustomed to, unemployment prior to cancer diagnosis.

A lot of people actually already low on the socioeconomic class [sic], they’re actually not working and then are just used to it. (Primary care focus group 3).

Despite the barriers identified for socio‐economically disadvantaged survivors, some participants felt barriers to these survivors’ return to work included reluctance or lack of incentive.

People in [lower SES metropolitan area], they’ve never gone back to work, so what they want is something that entitles them to freer benefits. (Primary care focus group 4).

In general, however, HCPs working in socio‐economically disadvantaged communities more often referred to a systemic lack of available support, rather than lack of individual motivation.

### 3.5 Systemic issues and solutions to remaining at/returning to work

Both survivors and HCPs frequently reported the need for factors affecting survivors’ employment to be addressed through system‐level changes, specifically education; resource availability and dissemination; legislation, including provisions for advocacy and representation; and procedures for HCPs to facilitate survivors’ remaining at/return to work.

Participants recommended ***education*** about the employment‐related needs of cancer survivors, both in terms of *workplace‐specific education*, and *public education* (including via *awareness campaigns* and *political leadership*).

Managers …need to be prepared and educated upfront that someone isn't going to be up to full speed for a while, whether it's reduced hours and more likely it would be reduced duties and that sort of thing. (Survivor interview 2).

Some participants also identified that *education of survivors* and *medical professionals* about (return to) work capacity, supports, and/or rights and responsibilities, could facilitate remaining at/returning to work.

Educate doctors on how to write graduated return to work [plans], medical certificates in the form of graduated return to work plans, because doctors just will say fit for 20 hours a week. They don’t know how to guide the patient and communicate with the workplace… you know they might be 20 hours but what type of duties? (Survivor interview 7).

Within the theme of ***resource availability***, *lack of resources or services* was highlighted as a hurdle in facilitating remaining at/returning to work and was frequently identified by focus groups of health professionals providing services for cancer survivors in disadvantaged circumstances. They noted that services for cancer survivors were *only available to some groups*, with services for those under 65 years of age being particularly difficult to access.

It’s hard, it’s just one of those things that the older ones have limited access to computers but their access to services is better. Whereas for the younger group, they can access internet information but there is very little services. (Primary care focus group 5).

Likewise, services were often only accessible to inpatients or depended on grant funding. A specific subtheme identified the *need for greater availability and access to allied health services*.

What I find is if – suppose this is somebody who does manual work, they’re needing an occupational therapist to look at that person and say are they able to do their work, but that’s expensive for patients. Most people aren’t under bulk billing unless you do some other way, probably allied health. (Primary care focus group 4).

Another subtheme identified the *need for more follow‐up care* in survivorship, noted by survivors and HCPs.

Several participants identified the need for *vocational rehabilitation or retraining*. Others considered that *access to volunteering roles* may be one way for survivors to maintain or develop skills relevant to employment.

Some participants reflected that better employment outcomes, including education and resource availability, would be best affected through ***changes in policy and legislation***. Several expressed that cancer survivors could have better employment outcomes if they had *advocacy and representation*, whether within the workplace or from an independent advocate. Others recommended *changes to legislation*, similar to Work Cover legislation (Australian legislation protecting those who injure themselves at work). Some indicated the need for changes to legislation concerning financial issues for cancer survivors such as *difficulty accessing superannuation* or *loss of pensions or benefits when returning to part time hours*.

### 3.6 Systemic facilitation of remaining at/returning to work by HCPs

Some participants felt that ***employment outcomes could be addressed on a systemic level via HCPs***.

So maybe we need to – and I'm sure it's been looked at before – but revisit how can doctors influence a more successful post cancer return to work. (Survivor interview 2).

However, while HCPs were happy to *encourage* remaining at/returning to work, some experienced *limitations in facilitating remaining at/returning to work on a systemic level*. For instance;

It’s not that I could give her an alternative employment that would be satisfying and fulfilling and safe, and you know, how do you advocate for her and advocate for those around her? (Treatment centre focus group).

However, HCP focus groups mentioned *involvement of a multidisciplinary team* or referral to allied health professionals such as occupational therapists, physiotherapists and social workers as potentially facilitating remaining at/returning to work for cancer survivors.

Finally, HCPs discussed the issue of *how to assess return to work capacity*. While some indicated little difficulty, most agreed there was *no specific tool*, nor had they received formal training in assessing return to work capacity in cancer survivors. Judgement of survivors’ capacity to perform employment‐related tasks was therefore subjective, with assessment consequently depending at times on an on‐going relationship with the patient and in‐depth knowledge of the requirements of their employment.

I am not prepared to put a percentage of capacity or incapacity. I haven't got the skills to do that. (Primary care focus group 2).

There’s basically no specific tools, it’s just based on experience, knowing the patients really well before, during and after. (Primary care focus group 3).

Some discussed a *multidisciplinary approach to return to work assessment*, including referral to other specialists such as occupational physicians. Despite this, GPs reported being responsible for completing much of the paperwork concerning return to work capacity.

## 4 DISCUSSION

This study aimed to identify barriers and facilitators of remaining at/returning to work for cancer survivors who were living with disadvantaged circumstances, whether due to regional/rural location or socio‐economic disadvantage. In doing so, it included perspectives of HCPs, with an emphasis on primary care professionals, and aimed to identify individual and systemic level issues in remaining at/returning to work for cancer survivors and recommendations for the targeting of these barriers.

Although our results were consistent with previous research and theory regarding the impact of personal or individual factors at the macro‐, meso‐ and micro‐levels on employment outcomes among cancer survivors,^15^ this study identified additional factors predominantly at the meso‐level, which are not included in previous research.^15^ Survivors and HCPs identified factors at the micro‐level such as demographic characteristics (eg age and proximity to retirement); meso‐levels (flexibility of employment and psychosocial support at work); and macro‐levels (economic situation and lack of policy or legal parameters to assess RTW capacity or implement flexible RTW strategies, community‐based support services).

Survivors from regional/rural communities were identified as experiencing significant additional barriers to employment predominately at the meso‐level. In Australia, where almost a third of population live in rural and remote locations,^35^ there are significant disparities in disease burden and health for those who live in outer regional, rural and remote areas compared with their metropolitan counterparts.^35^ It has also been well documented that the further one lives from a treatment centre the poorer the cancer outcome, although the mechanism responsible for this outcome is unclear.^36^ Our study offers some insight into understanding the additional strains placed on survivors seeking treatment for cancer. Participants identified a lack of local services, a need for travel to treatment and a disjuncture between treatment centres posing significant challenges for regional and remote cancer survivors when attempting to remain at/return to work, consistent with review findings indicating that travel to treatment causes practical and financial difficulty for rural survivors.^27^ Some support was found for the hypothesis that this disparity may in part be due to higher proportions of regional/rural employment involving manual labour.^19^ Further barriers included the lack of *any* available work and difficulty of employers in holding positions open for cancer survivors to return to. Positive aspects of regional and rural communities for cancer survivors looking to RTW included that rural communities and small employers were motivated to support cancer survivors in their community despite these difficulties, perhaps suggesting a greater sense of interpersonal connection and support. However, some felt that small communities led to lack of privacy and increased stigma.

Challenges identified for socio‐economically disadvantaged survivors included a lack of available employment and less flexible employment, consistent with previous findings indicating that inflexible work schedules are detrimental to employment outcomes;^37^ and with the suggestion that the more flexible working conditions potentially available to socio‐economically advantaged groups may partially explain relationships between socio‐economic advantage and higher employment.^20^

To our knowledge, this is the first Australian study to include perspectives of primary health‐care professionals working with disadvantaged communities, on barriers to remaining at/returning to work for cancer survivors. This is an important contribution considering the potential role of primary care in providing management of physical and psychological effects relevant to remaining at/returning to work for cancer survivors.^24^, ^26^ Participating HCPs recognized many of the same themes as survivors themselves, reflecting awareness of the challenges of facilitating remaining at/returning to work for cancer survivors. Discrepancies were found mainly in minor subthemes. HCPs discussed the impact of clinical disease characteristics and challenges with return to work assessment more extensively than survivors, while survivors spoke of returning to work due to motivation to ‘give back’, and rural survivors spoke of disjuncture from community‐level support, neither of which were explicitly discussed by HCPs. Inclusion of both perspectives therefore enabled a more thorough exploration of systemic issues affecting each group on remaining at/returning to work (survivors) or in supporting survivors to do so (HCPs).

HCPs tended to relate lack of employment availability and flexibility more specifically to socio‐economic disadvantage, whereas survivors who discussed these experiences tended not to explicitly refer to socio‐economic disadvantage even when the context suggested it (lower education of co‐workers, manual ‘blue collar’ work). This tendency may suggest survivor's focus on systemic issues (eg need for education), instead of self‐identifying with disadvantage. Some primary care professionals reflected that socio‐economically disadvantaged survivors may be accustomed to pre‐existing unemployment and saw these survivors as unmotivated to seek employment. The presence of these perspectives suggested the need for more understanding of social determinants of health and well‐being and systemic effects of socio‐economic disadvantage indicated in previous studies^16^, ^21^ and the need for systemic changes to address them. HCPs working in socio‐economically disadvantaged communities more often referred to a systemic lack of available support, rather than lack of individual motivation.

Participants’ identification of the need for system‐level changes (ie macro‐level) to achieve more positive employment outcomes for cancer survivors is consistent with reviews proposing the influence of policies, procedures and economic factors on other factors in return to work,^15^ and with reviews suggesting a role for policy change, including education on rights and obligations and better assessment of work capacities.^17^ Survivors and HCPs saw the need for changes to be implemented at societal and governmental levels, indicating the need for policy changes to address these issues. Frequently, a lack of resources and the need to integrate existing resources were identified as system‐level issues impacting on employment. These findings suggest the applicability of Feuerstein's cancer and work model to guide the identification and formulation of survivors needs at the micro‐, meso‐ and macro‐levels to prevent ‘long‐term work disability’ and potentially support return to work preferences of cancer survivors. In Australia, a greater focus on the factors at the meso‐level are required to address factors associated with geography and rurality factors each of which contribute to socio‐economic disadvantage and poorer cancer outcomes.

While this study achieved saturation of themes regarding barriers and facilitators in remaining at/returning to work for cancer survivors living with either geographic or socio‐economic disadvantage, it did not examine barriers and facilitators experienced by specific groups potentially living with disadvantage in Australia. Notably, previous research has indicated disparities in health and cancer outcomes for culturally and linguistically diverse (CALD) cancer survivors,^23^ and Indigenous Australian cancer survivors.^38^, ^39^ However, despite attempts, this study was not able to include the perspectives of either Indigenous cancer survivors or CALD cancer survivors. This difficulty indicates the need to design specific studies to examine employment issues for cancer survivors from these communities in more depth, as poorer health and cancer outcomes stand to impact on employment opportunities and experiences, which may then contribute to further disadvantage and isolation in these communities. Additionally, we chose to minimize detailed demographic data collection due to the challenges of recruiting and conducting research with targeted participants, including time limitations on primary care professionals, vulnerability of disadvantaged survivors who may be less likely to participate given additional burden, and potential concerns over identification in small communities; this decision meant we were unable to report age or individual‐level income. Finally, this study was intended as a qualitative exploration of survivor and HCP experiences of barriers and facilitators of remaining at/returning to work for cancer survivors from disadvantaged communities. Our methodology using one‐off participation in a focus group or interview allowed exploration of relevant issues while minimizing participation burden for health‐care professionals and survivors living with disadvantage. Future research may benefit from a prospective approach to following survivors’ experiences in attempting to remain at or return to work.

In summary, this study provided a rich account of barriers and enablers of remaining at or returning to work faced by Australian cancer survivors living in regional/rural and socio‐economically disadvantaged communities. Both HCPs and survivors recognized the need for these issues to be addressed at the system level, with education, legal representation and better provision of resources all identified as avenues for promoting greater health equity for these cancer survivors.

## CONFLICT OF INTEREST

All authors declare no conflict of interest.

## AUTHOR CONTRIBUTIONS

Vikki Knott, Paul Ward, Ian Olver, Julia Fallon‐Ferguson, Jon Emery, Chris Christensen, Monique Bareham and Bogda Koczwara made substantial contributions to conception and design of the study. Vikki Knott, Bogda Koczwara, Monique Bareham and Suzana Freegard collected the data. Suzana Freegard completed transcription of data. Emma Kemp completed the data analysis and interpretation with assistance from Vikki Knott and Suzana Freegard. All authors were involved in drafting the manuscript or revising it critically for important intellectual content. All authors gave final approval of the version to be published.

## Data Availability

The data that support the findings of this study are available on reasonable request from the corresponding author. The data are not publicly available due to privacy or ethical restrictions.

## APPENDIX 1 Topic guide—Supporting Return to Employment after Cancer in Disadvantaged Communities

*This topic guide has been created to provide you with an outline of the topics we aim to discuss during our session and to provide you with time to consider these. Feel free to make notes if you wish and bring them along to our focus group to remind you of your views in the group environment*

*In the session, we would like to explore your views on work before and after cancer treatment, barriers getting back to work and what resources/supports you think would be helpful assisting in you getting back to work after treatment. Your opinions will assist in the development of resources and practices to support return to work after cancer*.

***The following questions will be asked during your focus group*.**

1. Tell us a little about yourself.
2. What in your opinion are patients’ expectations regarding work after cancer before they commence cancer treatment?
3. How do you think they change, if at all, after they experience cancer treatment?
4. What is your view of the impact of cancer and its treatment on current and future work?
5. Are there any aspects to these experiences that are unique to patients living in rural/remote Australia?
6. Are there any aspects to these experiences that are unique to patients living in communities where employment is limited?
7. What do you see as the barriers to returning to work?
8. Who do you feel would be best to address these barriers? How could they help?
9. What supports and resources relating to employment would you like to have access to?
10. What would be the most useful format for these resources?

## APPENDIX 2 Themes in barriers and solutions in RTW for Australian cancer survivors living with disadvantage

**Table:**

Theme‐subtheme	Identified by:	Example quote
Individual‐level barriers/enablers: Cancer survivors in general		
Cancer‐related barriers/enablers		
Clinical disease characteristics	Consumers HCPs	‘Each cancer diagnosis is a different diagnosis. So even if it's, for example, breast cancer, you've got a whole range of different breast cancers. And the treatment is different for all of them’ (Primary focus group 5)
Symptoms and side‐effects	Consumers HCPs	‘I still have issues…with memory, fatigue, all those sorts of things…So, there's some side effects that have just not gone away. Like, they say it will get better. Some things don't. So that was very, very difficult. I lost my job after a few months’. (Paired survivor interview)
Psychological/emotional aftermath	Consumers HCPs	‘The other big thing that really sort of takes me back and I suspect it will probably with a lot of cancer survivors – and I know this is a direct result – is I’ve had issues with anxiety… I was never like this before’. (Survivor interview 1)
Survivor's mindset		
Work ethic	Consumers HCPs	‘I also have a very high work ethic and part of me wanted to keep working because ‐ well one I needed the money, but the other part was I didn't want to let people down. They've been very supportive to me’ (Survivor interview 2)
Pre‐existing RTW expectations	Consumers HCPs	‘I really believed I was going to be pretty much the same when I came out the other end…that I was just going to carry on as normal. But then I found that, the treatment being so complex, I needed a lot more time to recover’ (Survivor interview 3)
Maintaining normalcy	Consumers HCPs	‘We've got a young mum with breast cancer that's been successfully treated…once you have completed your treatment and you go, okay I’m not going to die tomorrow now and I’m going to…I want to have a normal life, I don't want to be defined by the fact that I had cancer’. (Primary focus group 5)
Change in priorities/outlook	Consumers HCPs	‘I just figured that if I only had finite time, and I was thinking only a couple of years at that time, I should enjoy what time I’ve got left and go into retirement because I didn't know how long I had left’. (Survivor interview 4)
‘Give back’ through work	Consumers	‘The nurses – of course the doctors were wonderful, but the nurses you see all the time and they were just so incredible, and I thought I want to [do that]’ (Survivor interview 5)
Sociodemographic barriers/enablers		
Age	Consumers HCPs	‘I thought, ‘Well, you know, I’m too young to retire so I think I’ll start up a part‐time business and work from home’. (Survivor interview 4)
Financial commitments	Consumers HCPs	‘Her issues ‐ her marriage has finished, they've separated, so she's the only source of income and she's trying to work’ (Primary care focus group 1)
Workplace‐related barriers/enablers		
Characteristics of work	Consumers HCPs	‘Particularly the breast cancer [patients]…the majority of them are middle class, professional women. I can think of a few that have manual jobs, but the majority of them have been able to get back to their professional employment. Some of them have taken longer than others depending on how protracted the treatment process has been’. (Primary care focus group 1)
Employer attitude	Consumers HCPs	‘I think depending on the…manager that you've got. You know, I’m in a [large organisation] that has a broad family/life balance policy. I have managers that are fairly strong in themselves as individuals, and so if you have all those factors, you're going to survive much better than in a situation that is a bit lesser than that’. (Survivor interview 6)
Co‐worker attitudes	Consumers HCPs	‘Other people who have gone back to work…they've had barriers from others about that they leave early, they have to go to appointments, therefore the others have to answer the phone, “Oh, you're always away. You're always at a doctor's appointment. How are you going? Are you okay?” but really simmering underneath because they've had to pick up extras’ (Survivor interview 4)
Communication with employers and co‐workers	Consumers HCPs	‘You've got some people that don't talk. I was quite open about it in my immediate work area. I felt I had to be honest with them because I just couldn't keep having all this time off work… but I was comfortable in doing that. But there are some people that aren't comfortable’. (Survivor interview 2)
Need for privacy	Consumers HCPs	‘It wasn't a good environment to return to, everybody knew what was going on’ (Survivor interview 6)
Flexibility in hours/leave	Consumers HCPs	‘I was never offered to come back part time or to come back as a test run and subsequently I guess I kept getting ill or very low in energy and suffering complications from the surgery that kept having me readmitted to hospital’. (Survivor interview 6)
Additional issues experienced by rural/remote survivors		
Need to travel long distances to treatment centres	Consumers HCPs	‘There's the burden of time, there's the burden of cost, there's the burden of being away from your loved ones, and for many people they're away for 6 weeks because PATs [payment scheme] won't help them to come back to [rural location] in between their cycles because they say it's not necessary’. (Primary focus group 2)
Disjuncture from community‐level support	Consumers	‘A lot of the time the patient would get diagnosed, they go to Adelaide for further specialist [appointments], and that's where we lose them…the ones that we're missing or slipping through the cracks, are the ones that go off to Adelaide for further information or have scans and things. Then they're not told’. (Consumer focus group)
Isolation from support services	Consumers HCPs	‘you know here in metro [areas] we have support groups, excellent, sharing experiences, especially going back to work. People learn so much from each other, and when you are in those rural and remote communities you really don't have access to that support’. (Treatment centre focus group)
Limited type/amount of work available		
Often requires physical/repetitive cognitive tasks	Consumers HCPs	‘It's generally smaller communities, less opportunities for certain variety of work types and things like that. So, if they've been a mechanic… to get back on the full job might be a very long process because it's such a physically demanding one. But to find an alternative position which isn't necessarily as demanding might be quite difficult in this community’. (Primary care focus group 3)
Farmers and self‐employed	Consumers HCPs	‘if you're self‐employed and you go off you've got ‐ your business still has to run. So, there you're thinking of work because you're running the business and keeping those clients’. (Primary care focus group 2)
Small businesses/communities		
Difficulty holding position open	Consumers HCPs	‘Be it farming or fishing or, you know, the commercial ventures down the street; the shops and what have you. They're all small. That would have been different, of course, if we were talking about a place that's bigger like here or the hospital…because there's ability to mop it up. But most places that people are employed [at] are very small employers’. (Primary care focus group 2).
Try to ‘look after their own’	Consumers HCPs	‘I think being small – even though we say some of these wineries and that are bigger employers they're still smaller country orientated places, they obviously look after their own’. (Primary care focus group 3)
Potential lack of privacy, discrimination	Consumers HCPs	‘I guess there's a few problems with disadvantaged communities…the stigma, and so people not wanting to say they've got cancer. They're often smaller communities, so everyone knows everyone, everyone knows everyone's business. So, it might be more difficult for people to you know access the services that might assist them with getting back to employment’ (Treatment centre focus group)
Large businesses impersonal	Consumers	‘I'm here but I'm not listening; some of the big companies. I mean, they're multi companies…They're big multinationals. There's only so many people they can carry’ (Consumer focus group)
Additional issues experienced by survivors living with socio‐economic disadvantage		
Higher unemployment	HCPs	‘You get that percentage that they have no jobs to go back to’ (Primary care focus group 5)
Less secure employment	HCPs	‘They've just started a new job, and they've suddenly had to start on the chemotherapy roundabout. They've got no sick leave accrued or they're casual’ (Treatment centre focus group)
‘Blue collar employers’ may be less understanding of RTW challenges	Consumers HCPs	‘If say it's an area where it's difficult to find a job and a lot of jobs are casual or I guess blue collar jobs. It might be more difficult to even I guess find understanding in that context than it would be for example if you worked in an area where you were with a bunch of health professionals’. (Treatment centre focus group)
Potentially accustomed to unemployment prior to diagnosis	HCPs	‘A lot of people actually already low on the socioeconomic class [sic], they're actually not working and then are just used to it’. (Primary care focus group 3)
Systemic issues and solutions		
Education		
Public education (political leadership; awareness campaigns_	Consumers HCPs	‘I think there could be far more education to general public about the impact that [cancer] has not only on the patient but then how it flows over onto the workplace when the person returns to work. And how one talks to someone else that's had to go through treatment’. (Survivor interview 6)
Workplace‐specific education/training	Consumers HCPs	‘I think it's education of your employers, and your work colleagues…. they might know you've gone through the journey that you have, but then your hair's growing back, you look fine…you seem fine. You look well. But they don't understand that you may still be on a treatment. Even though it's not chemotherapy it's still a treatment, it still affects things, and then there's the side effects, and unfortunately for me the prolonged side effects. So, I think a lot of it is education’ (Paired survivor interview)
RTW education for survivors	Consumers HCPs	‘They know about what supplements to take and whether to do yoga and meditation but that harder – ‘I don't know about rehab, what's rehabilitation’, because people don't know what vocational rehabilitation is’ (Survivor interview 7)
Education for HCPs on (facilitating) RTW	Consumers HCPs	‘Educating doctors about how to understand what a return to work involves, not to be too protective of the patient’ (Survivor interview 7)
Resource availability		
Lack of resources/services	Consumers HCPs	‘Then where do you refer someone for that support? Because you can't do it in ten minutes, you can't. So, people need to be able to access those sort of supports that they – once they've got through treatment to be able to make changes in their lives that they want’ (Primary care focus group 5)
Only available to some groups	HCPs	‘Once they're over 65 it's not so bad, you've got all sorts of different people that provide services to some degree but [for] the under 65s there's a massive gap…Financial support, any sort of things that you can get from any level that you would expect to support people going either back to work or back in life that is easily accessible in the over 65s, you can't be in the under 65s’. (Primary care focus group 5)
Need greater access to allied health	HCPs	‘Then you've got the whole issue of affordability again if someone takes off work and now they've got to go and source a psychologist or social worker or counsellor or something, they've got to find the funds for that and they might know that they need to talk to someone but if they can't afford it in the budget it becomes non‐essential doesn't it? Even though they might want it, you know?’ (Primary focus care group 5)
Need better follow‐up care for survivors	Consumers HCPs	‘The last treatment, ‘woohoo’, and I felt ridiculous. I felt absolutely abandoned and I think that would be something that needs to be addressed…given we are talking about returning to work after treatment, the psychological impact of that is huge’. (Survivor interview 2)
Vocational rehabilitation/training	Consumers HCPs	‘It's through rehabilitation that people address the barriers of returning to work. [There's] a lot of wasted work capacity sitting out there in people who aren't working, who do have a capacity for work if suitable duties can be designed and appropriate support mechanisms financially and practically in workplaces can be agree. So the employers feel confident they're not going to cause harm for their person, they're not going to stress them, or what can reasonably ask them, what they can reasonably ask them to do, and how far they're required to go making adjustments’ (Survivor interview 7)
Access to volunteering roles	Consumers HCPs	‘As [you] go through treatment and stuff, and then you're dismissed and you're made redundant, you become more withdrawn and depressed and stand back so part of the encouragement would be to consider being active in the community as a volunteer as a way of getting your skills back and becoming active again, if employment wasn't available’ (Survivor interview 4)
Legislative changes		
Advocacy/representation	Consumers HCPs	‘I mean, if they have a reason they can just say your position's been made redundant, and that's what they did. I think it's maybe having…a support department or…just someone from a legal point of view to coach you’. (Survivor interview 8)
Changes to legislation	Consumers HCPs	‘When I first looked at some of this return‐to‐work stuff, I thought the legislation needs to change, certainly with WorkCover [legislation … People who have got cancer or a long‐term chronic illness actually have the same barriers and discrimination but nothing to cover them’. (Survivor interview 4)
HCP facilitation of RTW	Consumers HCPs	‘Yeah I usually encourage them. So most men – they basically go back to work, I have two that still work in the vineyard and the treatment was basically successful, physically they're capable of doing the work and mentally they're capable of doing the same work, so just basically encouragement’. (Primary care focus group 3)
HCP limitations in facilitating RTW outside of health‐care system	Consumers HCPs	‘They don't deal with the wider system. So, an oncologist might say, “I want you to go back to work and leave it with me.” He doesn't want to know the details of all the problems of getting back to work, “My employer this and my employer that. I just can't do this. I can't do that’. (Survivor interview 4)
Involvement of MDT/allied health	HCPs	‘you'd probably involve a group of people. Obviously, you have the oncologist and then depending on what their requirement support is allied health would get involved or it might just be GPs’. (Primary care focus group 3)
How to assess RTW capacity	HCPs	‘in terms of saying, "This person has 70 per cent of their previous capacity," I would never make that assessment. I'd send someone to an occupational physician for that’. (Primary care focus group 2)
No specific tool	HCPs	‘There is no formal assessment. I'm not sure if there is any way of assessing how many too, I don't have a clue. I'm not sure whether occupational physician or therapists could be of some help. I don't know if they exist’. (Treatment centre focus group)
Multidisciplinary approach to RTW assessment	HCPs	‘The other thing that can happen is if we could have panels assessing to those with cancer, so the panel would have let's say a GP, a specialist, and an occupational therapist, a physiotherapist…,and as a group they make a decision, it's much easier because of all their expertise’. (Primary care focus group 4)
